# TSIS: an R package to infer alternative splicing isoform switches for time-series data

**DOI:** 10.1093/bioinformatics/btx411

**Published:** 2017-06-26

**Authors:** Wenbin Guo, Cristiane P G Calixto, John W S Brown, Runxuan Zhang

**Affiliations:** 1 Information and Computational Sciences, The James Hutton Institute, Invergowrie, Dundee, Scotland, UK; 2 Plant Sciences Division, School of Life Sciences, University of Dundee, Invergowrie, Dundee, Scotland, UK; 3 Cell and Molecular Sciences, The James Hutton Institute, Invergowrie, Dundee, Scotland, UK

## Abstract

**Summary:**

An alternative splicing isoform switch is where a pair of transcript isoforms reverse their relative expression abundances in response to external or internal stimuli. Although computational methods are available to study differential alternative splicing, few tools for detection of isoform switches exist and these are based on pairwise comparisons. Here, we provide the TSIS R package, which is the first tool for detecting significant transcript isoform switches in time-series data. The main steps of TSIS are to search for the isoform switch points in the time-series, characterize the switches and filter the results with user input parameters. All the functions are integrated into a Shiny App for ease of implementation of the analysis.

**Availability and implementation:**

The TSIS package is available on GitHub: https://github.com/wyguo/TSIS.

## 1 Introduction

Regulation of gene expression by alternative splicing (AS) generates changes in abundance of different transcript isoforms. One particular splicing phenotype is isoform switching where the relative abundance of different isoforms of the same gene is reversed in different cell types or in response to stimuli. Isoform switches often play pivotal roles in re-programming of gene expression and isoform switches of functionally different transcript isoforms between normal and tumor tissues provide signatures for cancer diagnostics and prognostics (Sebestyen *et al.*, 2015).

**Fig. 1:**
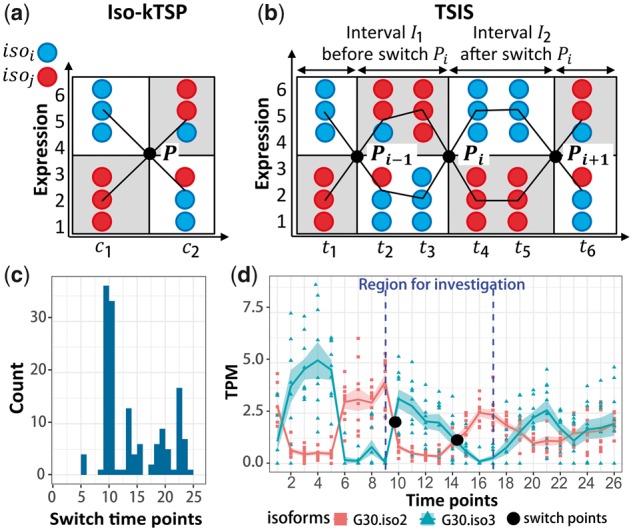
Analyzes of isoform switches. In (**a**) and (**b**), expression data with three replicates for each condition/time-point is simulated for isoforms $iso_{i}$ and $iso_{j}$. The points in the plots represent the samples and the black lines connect the average of samples. (a) A scheme plot for iso-kTSP that shows an isoform switch between two conditions $c_{1}$ and $c_{2}$. (b) A scheme plot for TSIS where two isoforms show three switches at different time-points. In (**c**) and (**d**), TSIS-generated output files are shown for real time-course RNA-seq data. (c) Histogram of isoform switches identified in 30 different genes. (d) Example of two transcript isoforms from gene G30 showing multiple switches, where user input parameter on the region for investigation has been labeled. TPM, transcripts per million

There are limited tools designed for inference of isoform switches and currently there is no software available for detecting alternative splicing isoform switches for time-series data. Isoform switch detection tools, such as iso-kTSP (Sebestyen *et al.*, 2015), spliceR (Vitting-Seerup *et al.*, 2014) and SwitchSeq (Gonzàlez-Porta and Brazma, 2014), only perform pairwise comparisons (Fig. 1a). Time-series RNA-seq data greatly enhances the resolution of changes in expression and AS during development or in responses to external or internal cues. Identification of isoform switches in time-series data presents specific challenges in that (i) switch points can happen between any time-points, and (ii) the isoform pairs may undergo a number of switches during the time course (Fig. 1b). To detect and characterize temporal and complex isoform switches, we developed the time-series isoform switch (TSIS) R package, which incorporates score schemes from current methods and includes a number of new metrics which capture the characteristics of the isoform switches.

## 2 Methods and application

TSIS detects pairs of AS transcripts with one or more isoform switches and genes with multiple pairs of transcripts which show isoform switches. By defining five metrics of the isoform switch, the method comprehensively captures and describes the isoform switches occurring at different points in time-series data. TSIS analysis can be carried out using command lines as well as through a graphic interface using a Shiny App (https://CRAN.R-project.org/package=shiny) where the analysis can be implemented easily.

### 2.1 Determine the switch points

We have offered two approaches to search for the switch points in TSIS. The first approach takes the average expression values of the replicates for each time-point for each isoform and searches for the cross points. The second approach uses natural spline curves to fit the time-series data for each transcript isoform using the R package ‘splines’ (version 3.3.2) and finds cross points of the fitted curves for each pair of isoforms. The spline method is useful to find global trends of time-series data when the data is noisy. However, it may lack details of isoform switches in the local region. It is recommended that users use both average and spline methods to search for the switch points and examine manually when inconsistent results were produced by the above two methods.

### 2.2 Define the switch metrics

The intersection points determined in Section 2.1 divide the time-series frame into intervals and each switch point is flanked by an interval before the switch and after the switch (Fig. 1b). We define the switch of two isoforms $iso_{i}$ and $iso_{j}$ by (i) the switch point $P_{i}$, (ii) time-points between switch points $P_{i-1}$ and $P_{i}$ as interval $I_{1}$ before switch $P_{i}$ and (iii) time-points between switch points $P_{i}$ and $P_{i+1}$ as interval $I_{2}$ after the switch $P_{i}$ (Fig. 1b). Each isoform switch is described by five metrics. Metric 1: $S_{1}$ represents the probability of the abundance switch and is calculated as the sum of the frequencies of two possible scenarios that one isoform is more or less abundant than the other in the two intervals adjacent to a switch point, as used in iso-kTSP (Sebestyen *et al.*, 2015).

$$S_{1}(iso_{i},iso_{j}|I_{1},I_{2})=|p(iso_{i}>iso_{j}|I_{1})+p(iso_{i}<iso_{j}|I_{2})-1|,$$

Where $p(iso_{i}>iso_{j}|I_{1})$ and $p(iso_{i}<iso_{j}|I_{2})$ are the frequencies/probabilities that the samples of one isoform is greater or less than in the other in corresponding intervals. Metric 2: $S_{2}$ is the sum of average abundance differences of the two isoforms in both intervals.

$$S_{2}(iso_{i},iso_{j}|I_{1},I_{2})=d(iso_{i},iso_{j}|I_{1})+d(iso_{i},iso_{j}|I_{2})$$

Where $d(iso_{i},iso_{j}|I_{k})$ is the average difference of abundances between $iso_{i}$ and $iso_{j}$ in interval $I_{k},k=1,2$ defined as

$$d(iso_{i},iso_{j}|I_{k})=\frac{1}{\left|I_{k}\right|}\sum_{m_{I_{k}}}\left|\;\text{exp}(iso_{i}|s_{m_{I_{k}}},I_{k})-\text{exp}(iso_{j}|s_{m_{I_{k}}},I_{k})\right|$$

$\left|I_{k}\right|$ is the number of samples in interval $I_{k}$ and $\text{exp}(iso_{i}|s_{m_{I_{k}}},I_{k})$ is the expression of $iso_{i}$ of sample $s_{m_{I_{k}}}$ in interval $I_{k}$. Metric 2 indicates the magnitude of the switch. Higher values mean larger changes in abundances before and after the switch. Metric 3 measures the significance of the differences between the isoform abundances before and after the switch using paired *t*-tests to generate *P*-values for each interval. Metric 4 is a measure of whether the effect of the switch is transient or long lived (reflecting the number of time-points in the flanking intervals). Metric 5: Isoforms with high negative correlations across the time-points may identify important regulation in alternative splicing. Thus we also calculated the Pearson correlation of two isoforms across the whole time-series.

### 2.3 Filter and visualize the results

TSIS provides histograms that show the number of switches happening at each time-point as well as interactive visualizations of the isoform switch profiles (Fig. 1c, d). TSIS also allows regions of interest to be defined (Fig. 1d) or switches involving the most abundant isoforms or any predefined list of isoforms to be selected as outputs. Known IS in *Arabidopsis* circadian clock genes AT1G01060 (G2), AT5G37260 (G29) and AT3G09600 (G12) (Fig. 1c) (Filichkin *et al.*, 2015; James *et al.*, 2012a, 2012b) were successfully detected by TSIS. The example dataset (used in Fig. 1c, d) and details to run the tool are shown in the user manual on the Github page.
